# Does disrespect and abuse during childbirth differ between public and private hospitals in Southeast Nigeria

**DOI:** 10.1186/s12884-021-04298-z

**Published:** 2021-12-31

**Authors:** Ijeoma Nkem Okedo-Alex, Ifeyinwa Chizoba Akamike, Irene Ifeyinwa Eze, Chika Nwamma Onwasigwe

**Affiliations:** 1 Department of Community Medicine, Alex Ekwueme Federal University Teaching Hospital Abakaliki, Abakaliki, Ebonyi State Nigeria; 2 African Institute for Health Policy and Health Systems, Ebonyi State University, Abakaliki, Nigeria

**Keywords:** Respectful maternity care, Disrespect and abuse during childbirth, Mistreatment during childbirth, Intrapartum care, Facility-based childbirth, Quality of care, Nigeria

## Abstract

**Background:**

Disrespect and Abuse (D&A) during childbirth represents an important barrier to skilled birth utilization, indicating a problem with quality of care and a violation of women‘s human rights. This study compared prevalence of D&A during childbirth in a public and a private hospital in Southeast Nigeria.

**Methods:**

This study was a cross-sectional study among women who gave birth in two specialized health facilities: a public teaching and a private-for-profit faith-based hospital in Southeast Nigeria. In each facility, systematic random sampling was used to select 310 mothers who had given birth in the facility and were between 0-14 weeks after birth. Study participants were recruited through the immunization clinics. Semi-structured, interviewer-administered questionnaires using the Bowser and Hills classification of D&A during childbirth were used for data collection. Data were analyzed using SPSS version 20 at 95% significance level.

**Results:**

Mean age of the participants in the public hospital was 30.41 ± 4.4 and 29.31 ± 4.4 in the private hospital. Over three-fifths **(**191; 61.6%) in the public and 156 women (50.3%) in the private hospital had experienced at least one form of D&A during childbirth [cOR1.58; 95% CI 1.15, 2.18]. Abandonment and neglect [Public153 (49.4%) vs. Private: 91 (29.4%); cOR2.35; 95% CI. 1.69, 3.26] and non-consented care [Public 45 (14.5%) vs. Private 67(21.6%): cOR0.62; 95% CI. 0.41, 0.93] were the major types of D&A during childbirth. Denial of companionship was the most reported subtype of D&A during childbirth in both facilities [Public 135 (43.5%) vs. Private66 (21.3%); cOR2.85; 95% CI. 2.00, 4.06]. Rural residents were less likely to report at least one form of D&A during childbirth (aOR 0.53; CI 0.35-0.79).

**Conclusion:**

Although prevalence was high in both facilities, overall prevalence of D&A during childbirth and most subtypes were higher in the public health facility. There is a need to identify contextual factors enabling D&A during childbirth in public and private health care settings.

**Supplementary Information:**

The online version contains supplementary material available at 10.1186/s12884-021-04298-z.

## Background

In every country and community worldwide, pregnancy and childbirth are significant events in the lives of women and their families. Every woman has the right to the highest attainable standard of health, which includes the right to dignified, respectful health care throughout pregnancy and childbirth, as well as the right to be free from violence and discrimination [1, 2].The World Health Organization’s (WHO’s) quality of care framework for maternal and newborn health (MNH) care contains eight domains, three of which relate to women’s experience of care and include effective and responsive communication, care provided with respect and dignity and social and emotional support of the woman’s choice [3, 4]. While disrespect and abuse (D&A) of women may occur throughout pregnancy, childbirth and the postpartum period, women are particularly vulnerable during childbirth. Such practices may have direct adverse consequences for both mother and newborn [2]. Women who have previously been treated disrespectfully during childbirth and those hearing from their experiences may avoid facility childbirth, even if they have complications. This may contribute to increased maternal and perinatal mortality and morbidity. Such avoidable complications include obstructed labour, birth asphyxia, severe bleeding after childbirth, post-childbirth maternal and neonatal infections in addition to the negative psychological effects on women [5–7**]**.D&A during childbirth can act as a more powerful deterrent to current and/or future skilled birth care than any other more commonly recognized deterrent such as geographic and financial obstacles [8]. According to the 2018 Nigerian Demographic and Health Survey (NDHS), only 39% of births by Nigerian women occurred in health facilities and 43%were assisted by skilled birth attendants. Unskilled providers such as traditional birth attendants and relatives/friends assisted in 42% of births, while 11% had no external assistance during childbirth [9]. Although this national data did not explore D&A during childbirth, studies have found that D&A undermined utilization of health facilities for births and encouraged traditional birth attendance [10, 11].

In 2010, Bowser and Hill described in a landscape analysis seven categories of D&A during childbirth in facilities: physical abuse, non-consented clinical care, non-confidential care, non-dignified care, discrimination, abandonment/denial of care and detention in health facilities.^8 ^D&A has further been re-conceptualized as mistreatment during childbirth which is inflicted not only by individual providers, but also by health systems as a whole when conditions in facilities deviate greatly from accepted standards of care in infrastructure, staff requirements, equipment and supplies needed to provide that care [7, 12].

Globally, some studies have shown that non-use or delayed use of health facilities for childbirth have been reported due to poor quality of care and D&A received in health facilities alongside other social determinants of health [13–17]. Occurrence of D&A during childbirth has been explored by mostly qualitative studies, but few have used a mixed-methods approach to compare experiences of women in public and private health facilities **[**7, 10, 15, 18–27]. The objective of this study was to compare prevalence and forms of D&A during childbirth in public and private specialized healthcare settings in Southeast Nigeria.

## Methods

### Study Area

The study was conducted in two hospitals offering specialized care: a private-for-profit specialist hospital located in Afikpo North Local Government Area and a public tertiary hospital located in Abakaliki Local Government Area, both in Ebonyi State, Nigeria. These two hospitals were selected based on their obstetric work load and patronage. Both hospitals are referral facilities with patronage from urban and rural areas within the State. On a monthly basis, an average of 85 births occurs in the private hospital while the public hospital records 150 births.

The private-for-profit health facility is a specialist faith-based hospital. Clinical care in this hospital includes obstetrics and gynecology, pediatrics, surgery, internal medicine, general outpatient care and accident and emergency care. Recently, the hospital began residency programs for family medicine and obstetrics and gynecology. The immunisation clinic holds once a week with an average number of 100 children per clinic day.

The public health facility is the only teaching hospital in Ebonyi State. Some departments are obstetrics and gynaecology, paediatrics, internal medicine, surgery, community medicine and the adult accident and emergency unit. The immunization clinic holds twice a week and has about 80 children per clinic day.

### Study Design and Population

This study was a comparative cross-sectional study among women who attended child immunization clinics fourteen weeks or less after childbirth in the two health facilities.

Minimum sample size was calculated with a power of 80%, an anticipated non-response rate of 10% and a significance level of 5% at 299 women per hospital [28]. A total of 620 women, 310 participants per hospital, was selected for the study.

Systematic random sampling was used to select the study participants. Duration of recruitment was 10 weeks per facility. Using the immunization clinic registers, the sampling interval (K) was calculated by dividing average number of attendees by the number of study participants to be recruited that day. Simple random sampling technique (ballot method) was used to select the first participant in both hospitals [28, 29]. When the selected participant using the systematic scheme was not eligible, she was dropped and the next eligible participant recruited until the desired sample size was realized. Non-eligibility was defined as: childbirth not in the two hospitals and >14 weeks after birth.

### Data Collection Instruments

The instrument for this study was a pre-tested semi-structured interviewer-administered questionnaire adapted from a previous study [18]. The first section of the questionnaire was used to collect information on socio-demographic characteristics, including age, marital status, religion, place of residence, occupation and educational level. The second section collected information on the seven Bowser and Hill categories of D&A during childbirth: physical abuse (5 questions), non-consented care (7 questions), non-confidential care (6 questions), non-dignified care (5 questions), discriminatory care (6 questions), abandonment of care (5 questions) and detention in the health facility (3 questions) [8]. D&A during childbirth was said to have occurred if the participant answers yes to any of the specific questions in the questionnaire.

### Data Collection Methods

Data collection was carried out by the researcher and five trained research assistants. The assistants were resident doctors in Community medicine, who are uninvolved in the continuum of care of the women. They were trained on a brief overview of D&A during childbirth and different types, specific objectives of the study and how to administer the questionnaire. Training lasted for three hours. Pre-testing of the questionnaire was done among women (5% of the sample size) who met the inclusion criteria in another health facility in Abakaliki. Issues detected during pre-testing such as rephrasing of questions and typographic errors were addressed. A reliability test, using data from the pre-testing, yielded a Cronbach’s alpha value of 0.818. The questionnaires were administered in the health facility and took approximately seven minutes to fill.

### Measurement of variables

Socio-demographic characteristics (age, marital, educational and employment status, place of residence, religion, socio-economic status) and duration between childbirth and date of the survey were independent variables. STATA statistical software version 12 [30] was used to develop the socio-economic index, using Principal Component Analysis (PCA). Input to the PCA included information on estimated monthly family income, ownership of eight household items that included television, generator, microwave, electric pressing iron, fridge, gas cooker, car and air conditioner. For calculation of distribution cutting points, quartiles (Q) were used as Q1 poorest, Q2 the very poor, Q3 the poor and Q4 least poor [31]. This was further dichotomized into low socio-economic class (Q1- Q2) and high socio-economic class (Q3-Q4). Each participant was assigned a wealth index score of the household.

Dependent variables were prevalence of at least one type of D&A during last childbirth and different forms of D&A. Prevalence of D&A during childbirth was assessed using ‘Yes’ and ‘No’ questions on ever experiencing any form or category of D&A. Each question under the different categories of D&A during childbirth was assessed with binary options (Yes’ and ‘No). The proportion of participants who answered ‘Yes’ to any of the forms of D&A under each category was taken as the prevalence of experiencing D&A during childbirth. The proportion of participants who answered ‘Yes’ to any of the questions in the seven sub-categories of D&A, was used to compute the prevalence of each category and form of D&A during childbirth.

### Data analysis

A computer-based Statistical Package for Social Sciences (SPSS) for Microsoft Window version 20 software [32] was used for data analysis. Frequency tables were used for descriptive statistics of the variables and relevant means, standard deviations and proportions were calculated. Means and range of D&A during childbirth were calculated.

Frequencies and proportions were calculated for categorical variables while means and standard deviations were calculated for numeric/quantitative variables. Proportions of socio-demographic characteristics, prevalence of at least one form, categories and sub-categories of D&A during childbirth were compared between the women in the public and private hospitals using chi-square statistics at 5% level of significance while mean age was compared using student t-test (difference of two means) at the same level of significance. Chi square statistics were also used to assess the relationship between prevalence of at least one form of D&A during childbirth and socio-demographic characteristics. Independent variables with *p*<0.20 were included in a multivariable logistic regression model to determine predictors of the occurrence of at least one form of D&A during childbirth.

## Results

Mean age of the participants in the public hospital was higher than that in the private hospital (30.41 ± 4.4 vs. 29.31 ± 4.4; *p* = 0.002). More participants from the public hospital had post-secondary education (81.9%) than those in the private hospital (60.0%) (*p* = 0.001). A greater proportion of participants in the public hospital were paid employees compared to their private hospital counterparts (55.8% vs. 44.8%; *p* = 0.005). A higher proportion of participants in the public hospital belonged to the upper socioeconomic quartile (37.7% vs. 12.3%; *p* < 0.001) (Table 1).

**Table 1:** Socio-demographic and household characteristics of the participants

Variable	Private hospital *n*=310 (%)	Public hospital *n*=310 (%)	***p value***
**Age (years)**			0.090
15-24	31 (10.0)	18 (5.8)	
25-34	232 (74.8)	233 (75.2)	
35-44	47 (15.2)	59 (19.0)	
**Mean age (mean ±SD)**	29.31 ± 4.4	30.41 ± 4.4	0.002*
**Marital status**			
Currently married	283 (91.3)	302 (97.4)	0.001*
Currently unmarried^	27 (8.7)	8 (2.6)	
**Educational level**			
No formal Education	1 (0.3)	1 (0.3)	
Primary education	9 (2.9)	3 (1.0)	<0.001*
Secondary education	114 (36.8)	52 (16.8)	
Post-secondary education	186 (60.0)	254 (81.9)	
**Religious Denomination**			
Catholic	153 (49.4)	145 (46.8)	0.819
Anglican/Protestant	53 (17.1)	51 (16.4)	
Others	6 (1.9)	5 (1.6)	
Pentecostal	98 (31.6)	109 (35.2)	
**Employment status**			
Unemployed	47 (15.2)	51 (16.5)	0.005*
Self-employment	124 (40.0)	86 (27.7)	
Paid employment	139 (44.8)	173 (55.8)	
**Place of Residence**			
Rural	123 (39.7)	31 (10.0)	<0.001*
Urban	187 (60.3)	279 (90.0)	
**Duration post-childbirth**			
0-6 weeks	168(54.2)	131(42.3)	0.003*
7-14 weeks	142(45.8)	179(57.7)	
**Socioeconomic quartiles**			
1^st^ Quartile (Poorest)	126 (40.6)	29 (9.4)	<0.001*
2^nd^ Quartile (Very poor)	87 (28.1)	69 (22.3)	
3^rd^ Quartile (Poor)	59 (19.0)	95 (30.6)	
4^th^ Quartile (Least poor)	38 (12.3)	117 (37.7)	

*statistical significance +t-test

Overall, most different forms and types of D&A during childbirth were very frequent in both hospitals. The highest prevalence of D&A forms in both facilities were being beaten, slapped or pinched [public23 (7.4%) vs. private19 (6.1%)], episiotomy without consent [public 12 (5.5%) vs. private 23 (7.4 %)], and augmentation of labour without consent [public 24 (7.7 %) vs. private 16 (5.2 %)]. In the public hospital, less women 4 (1.3%) had their pubic hair shaved without consent than in the private hospital 18 (5.8%) [cOR 0.21; 95% CI. 0.07, 0.63] (Table 2).

**Table 2:** Prevalence of physical abuse, non-consented, non-confidential and discriminatory care during childbirth

Variable	Public hospital (*n*=310)	Private hospital (*n*=310)	Crude Odds ratio	95% CI
	Yes (%)	Yes (%)		
**Physical Abuse**				
Episiotomy given or sutured without anaesthesia	12(3.9)	22(7.1)	0.53	0.26-1.09
Beaten, slapped or pinched	23(7.4)	19(6.1)	1.16	0.62-2.16
Tied down or restrained during labour	6 (1.9)	10(3.2)	0.59	0.21-1.65
Other ways of physical abuse^b^	2 (0.6)	6 (1.9)	0.33	0.07-1.64
Sexually abused by health worker during labour	0 (0.0)	3 (1.0)	^e^	^e^
**Non-consented care**				
Episiotomy without consent	12(5.5)	23(7.4)	0.72	0.38-1.38
Shaving of pubic hair without consent	4 (1.3)	18(5.8)	0.21	**0.07-0.63**^a^
Augmentation of labour without consent	24(7.7)	16(5.2)	1.54	0.80-2.96
Caesarean birth without consent	7 (2.3)	16(5.2)	0.43	0.17-1.05
Blood transfusion without consent	3 (1.0)	10(3.2)	0.29	0.08-1.08
Sterilization without consent	3 (1.0)	7 (2.3)	0.42	0.11-1.65
Other services without consent^c^	3 (1.0)	8 (2.6)	0.37	0.09-1.40
**Non-confidential care**				
Provision of care without privacy	4 (1.3)	8 (2.6)	0.49	0.15-1.66
Medical history disclosure without consent	1 (0.3)	7 (2.3)	0.14	0.02-1.15
Age disclosure without consent	2 (0.6)	6 (1.9)	0.34	0.07-1.64
Disclosure of child’s paternity without consent	2 (0.6)	5 (1.6)	0.40	0.08-2.06
Disclosure of HIV status without consent	0 (0.0)	4 (1.3)	^e^	^e^
Other forms of non-confidential care^d^	1 (0.3)	4 (1.3)	0.25	0.03-2.23
**Discriminatory care**				
Denial of needed attention because of low social class or socio-economic status	1 (0.3)	4 (1.3)	0.25	0.03-2.23
Denial of needed attention because of teen age (≤ 19 years)	0 (0)	2 (0.6)	^e^	^e^
Denial of needed attention because of single motherhood status	1 (0.3)	2 (0.6)	0.49	0.05-5.53
Denial of needed attention because of HIV-seropositive status	0 (0)	2 (0.6)	^e^	^e^
Denial of needed attention because of low educational status	3 (1.0)	1 (0.3)	3.02	0.31-29.19
Denial of needed attention on the basis of ethnicity	1 (0.3)	0 (0.0)	^e^	^e^
Other reasons for discriminatory care	1 (0.3)	0 (0.0)	^e^	^e^

^a^ Statistical significance ^b^shoving and handling roughly ^c^Intravenous access and vaginal examination without consent ^d^Discussion of medical history and clinical examination with/in the presence of medical students ^e^Odds ratio was not calculable for variables with empty cells.

A lower proportion of women experienced non-consented care in the public hospital 45 (14.5%) compared to their private hospital counterparts 67 (21.6) [cOR 0.62; 95% CI. 0.41, 0.93] (Table 3). The proportion of women who reported at least one form of D&A in the public hospital was significantly higher [Public 191 (61.6%) vs. Private 156 (50.3%): cOR 1.58; 95% CI. 1.15, 2.18] Abandonment and neglect was reported by 153 (49.4%) of women in the public hospital compared to 91 (29.4%) in the private hospital [cOR 2.35; 95% CI. 1.69, 3.26] (Table 3).  Denial of companionship by the hospitals was the most reported subtype of D&A during childbirth in both facilities and this was higher in the public health facility [Public 135 (43.5%) vs. Private 66 (21.3%): cOR 2.85; 95% CI. 2.00, 4.06]. In the public hospital, fewer women were detained after childbirth because of inability to pay up bills for themselves [Public 8 (2.6%) vs. Private 21 (6.8%): cOR 0.37; 95% CI. 0.16, 0.84] as well as for the baby [Public 4 (1.3%) vs. Private 14 (4.5%): cOR 0.28; 95% CI. 0.09, 0.85]. There were no differences in the mean number of D&A during childbirth between the two facilities (Public1.04 ± 1.12vs. Private1.00 ± 1.33; *P* = 0.613) (Table 3).

**Table 3:** Prevalence of abandonment/neglect, non-dignified care and detention following childbirth and at least one form of D&A during childbirth

Variable	Public hospital (*n*=310)	Private hospital (*n*=310)	Odds ratio	95% CI
	Yes (%)	Yes (%)		
**Abandonment/Neglect**				
Denied companionship in labour	135(43.5)	66 (21.3)	2.85	**2.00-4.06**^a^
Left alone unattended in second stage of labour	23 (7.4)	17 (5.5)	1.38	0.72-2.64
Birth attendant failed to intervene or call for help from experienced staff in life threatening conditions	12 (3.9)	13 (4.2)	0.92	0.41-2.05
Not granted requested attention because staff was exhausted	12 (3.9)	11 (3.5)	1.10	0.48-2.52
Abandoned/neglected for other reasons	15 (4.8)	7 (2.3)	2.20	0.89-5.48
**Non-dignified care**				
Threatened with Caesarean section or poor pregnancy outcome in order to discourage women from shouting in labour	30 (9.7)	31 (10.0)	0.96	0.57-1.64
Scolded, shouted at or called stupid while in labour	36 (11.6)	27 (8.7)	1.38	0.81-2.33
Received slanderous remarks (aspersions) from birth attendants during labour	21 (6.8)	20 (6.5)	1.05	0.56-1.99
Blamed or intimidated during childbirth	13 (4.2)	16 (5.2)	0.80	0.38-1.70
Other forms of non-dignified care"	4 (1.3)	4 (1.3)	1.00	0.25-4.04
**Detention in the health facility following childbirth**				
You could not pay up your hospital bills at discharge	8 (2.6)	21 (6.8)	0.37	**0.16-0.84**^a^
You could not pay up baby’s hospital bills at discharge	4 (1.3)	14 (4.5)	0.28	**0.09-0.85**^a^
Other reasons^c^	4 (1.3)	3 (1.0)	1.34	0.30-6.03
**At least one form of D&A during childbirth**	191(61.6)	156(50.3)	1.58	**1.15-2.18**^a^
**Categories of D&A during childbirth**				
Abandonment and neglect	153(49.4)	91 (29.4)	2.35	**1.69-3.26**
Non-consented care	45 (14.5)	67 (21.6)	0.62	**0.41-0.93**^a^
Non-dignified care	61 (19.7)	57 (18.4)	0.62	**0.41-0.93**^a^
Physical abuse	39 (12.6)	45 (14.5)	0.85	0.53-1.34
Detention	12 (3.9)	25 (8.1)	0.46	**0.23-0.93**^a^
Non-confidential care	9 (2.9)	16 (5.2)	0.55	0.24-1.26
Discriminatory care	6 (1.9)	7 (2.3)	0.85	0.28-2.57
Mean number of D&A	1.04 ± 1.12	1.00 ± 1.33		
Maximum number of D&A	6	5		
Minimum number of D&A	0	0	-	

^a^ Statistical significance D&A: Disrespect and Abuse” Threatened with abandonment to discourage uncooperativeness and rudeness to birth companions/relatives ^c^Detained on account of unsatisfactory health status of mother and/or child which mother perceived as satisfactory and/or a guise for increased hospital bills

In the multivariable logistic regression model, women who resided in the rural areas had 47% lower odds of reporting at least one form of D&A during childbirth (aOR 0.53; CI 0.35-0.79) (Table 4).

**Table 4:** Predictors of at least one form of D&A during childbirth (*n*=620)

Variable	adjusted Odds Ratio	95% CI	
		Lower	Upper
**Employment status**			
Unemployed	1.46	0.93	2.30
Employed	1		
**Place of Residence**			
Rural	0.53	0.35	0.79
Urban	1		
**Socioeconomic status**			
Low socioeconomic status	1.09	0.76	1.56
High socioeconomic status	1		
**Duration after birth**			
0-6 weeks	0.87	0.63	1.21
7-14 weeks	1		
**Birthing facility type**			
Private	0.74	0.52	1.06
Public	1		

## Discussion

### Principal findings

More women had post-secondary education and belonged to the uppermost wealth quartile in the public hospital rather than those in the private one. This finding could be because in the context of the study area, teaching hospitals (the public hospital in this study) are well known to offer a wider range of specialist care in addition to employing higher numbers of skilled health workers and these may be better appreciated and sought after by those who are more educated and can better afford this care because they are in the upper wealth quartiles. However, the population in these two hospitals may be different from the distribution of education and wealth quartiles in the general population in Nigeria [9, 33].

The prevalence of abandonment and neglect during childbirth was the major category of D&A experienced in both hospitals. These were all statistically significantly higher in the public hospital as compared to the private hospital. The difference in prevalence of abandonment/neglect during childbirth between the public and private hospital could be because the public hospital is a bigger referral hospital (the only teaching hospital in the State) and may also have more women referred for complications than the private hospital. This could overwhelm the ability of the staff leading to perceived feelings of being abandoned. On the other hand, staff may become more friendly with women who have complications than with those who have no complications. Consistent with our findings, abandonment was among the most reported forms of D&A during childbirth in other studies [19, 23, 34–36]. This high prevalence of abandonment is particularly worrisome because the assured availability of a health provider during childbirth is an aspect of care considered important by women who have passed through the childbirth process [37]. Under this abandonment category, being denied companionship in labour by the husband or close relatives was most often reported. Companionship during labour and childbirth have been found to improve maternal mental wellbeing and obstetric outcomes [38]. Nonetheless, implementation of this effective and affordable intervention has remained suboptimal in many settings such as ours [38, 39]. On the other hand, non-consented and non-dignified care were most commonly reported in other studies [18, 40].

In contrast to our findings, other studies (also in teaching hospitals) found higher proportions of non-consented care during childbirth (54%-100%) **.** An explanation could be that women in those studies were interviewed earlier after birth than in ours. Findings from other studies are consistent with the different forms of non-consented care in our study [18, 23, 34, 41]. In line with ethical best practices, informed consent is supposed to be obtained from women before any procedure is carried out. These reports of non-consented care are a breach of ethical principles and highlight the need to improve ethics-based obstetric practices. One reason for this could be that in resource–limited environments, litigations related to such matters are relatively uncommon and the implicit assumption that health providers are to make decisions regarding maternity care for women with or without their informed consent [18, 42].

Non-dignified care was also commonly reported in this study however more respondents (29.6%) experienced non-dignified care in another Nigerian study [18], while it was the commonest type of D&A meted out to women during childbirth in Mali [43].

Discriminatory care during childbirth has been documented in our and other studies [18, 44]. Discriminating against a parturient for any reason breaches the fundamental health rights of women. Thus there is need for continuous training and supervision of obstetric healthcare providers in order to ensure non-discrimination in patient care.

The prevalence of at least one form of D&A during childbirth was high in both hospitals, but significantly higher in the public hospital.

Women were interviewed in the health facility where their childbirth had occurred and this could have introduced courtesy bias. It has been suggested, however, that a longer period after birth may allow time for mental processing the birth experience and thus improve recall. Alternatively, a different school of thought opines that women may tend to forget the nasty birth experiences the longer the time passed between the experience and the survey [45]. A study among women in Ethiopia, Kenya, Madagascar, Rwanda and Tanzania reported a prevalence of 60% of overall violations of respectful maternity care rights following objective assessment using a structured checklist [19]. Observation of women-provider interactions by trained observers in an Ethiopian study found a prevalence of 74.8%, nonetheless, only 22% of the women reported D&A during childbirth [23]. Normalization of D&A during childbirth by mothers in the study may have made them fail to consider and/or report certain disrespectful practices leading to the incongruence between observed and self-reported events. Objective assessment of D&A by trained observers using structured checklists have shown to provide higher prevalence estimates of D&A during childbirth than subjective reporting [46]. Self-reported prevalence of D&A during childbirth in our study may thus have been underestimated. A review of D&A during childbirth in Ethiopia and sub-Saharan Africa found a pooled prevalence of 44%-49.4% [35, 36]. Beyond being pooled estimates, the lower prevalence in these reviews may be because some of those studies were community-based and involved women at different points in time after birth.

Disparities that could account for the prevalence of at least one form of D&A in both hospitals include the fact that private hospitals, especially mission hospitals, tend to have more stringent measures in place to ensure respect for women. This includes close supervision of staff and oversight of the facility by the church, ready patient’s access to hospital or church management for complaints of disrespectful care and follow-up of such complaints in addition to the moralistic undertone with which care is rendered in private hospitals. Also, such hospitals may attract patronage from faithful people who may have more religion-related acceptance for provided health care. In contrast, bureaucratic processes, poor ownership attitudes by workers and loose redressing mechanisms have been associated with public hospitals [47, 48].

Our findings portray the need to improve maternity experiences of parturient and build an institutional culture of respectful care. This will include systematic identification of underlying contributors to D&A during childbirth as well as designing and implementing context-specific interventions to address these in the two facilities. Such interventions may involve development of institutional indicators of D&A during childbirth, their routine evaluation and using lessons learned to improve maternity experiences of women during childbirth. It is equally important to institutionalize continuous provider training, mentorship, monitoring and supportive supervision that build interpersonal communication skills aimed at promoting woman-centred care in both public and private health care settings. Such training can be integrated into existing facility-based continuous educational platforms such as grand rounds, professional group seminars and morning reviews. This is in addition to the use of provider-independent mechanisms and checks such as automated and administrative processes that routinize procedures such as informed consent before clinical care is provided. An emerging nascent area of focus from our study is the need for cross-learning and collaboration between the public and private health sectors, all geared towards improving maternal experiences of maternity care.

Previous studies have shown that women who resided in rural areas reported less D&A during childbirth than those residing in urban areas, in line with our findings [49, 50]. In other studies, being older than 19 years, having no formal education, at least ninth grade and secondary education, self-reported depression, absence of support persons during childbirth, longer labour duration, and birth via Caesarean section were more likely to experience D&A during childbirth while parity (more than four births) reduced the likelihood [49–51].

Women who reside in rural areas tend to be poorer, less educated, less equipped with materials for childbirth, less aware of their rights and thus more prone to experience D&A during labour and childbirth without recognizing it. Structural gender inequities evidenced by paucity of information, lack of financial stability and autonomy to exercise rights contribute to perpetuating D&A during childbirth [52]. Additionally, rural women may be more inclined to consider such ‘mistreatments’ as normal given culture-related patriarchal settings of rural African communities and expected subservience of women [53]. Their urban counterparts may be more knowledgeable about their rights and have higher expectations regarding their healthcare experiences with more openness in sharing D&A during childbirth. These findings highlight the need for intersectoral collaboration targeted at improving the status of women through female education, financial empowerment, awareness of rights and support for justice.

Some areas for future research would include the use of observations in estimating the prevalence and isolating enablers of D&A during childbirth from the perspectives of women and health providers in a larger number of public and private hospitals. Future research is also needed to develop and assess the effectiveness of contextually relevant interventions to reduce D&A during childbirth in both public and private health care settings.

### Strengths and limitations

This is one of the few studies in Nigeria that have estimated prevalence of D&A during childbirth with quantitative methods. More so, to the best of our search, this is one of the first studies to compare experiences of D&A in public and private health care settings. Lastly, this study utilized a fairly large sample. Some limitations include the fact that it was conducted in only two facilities which were non-randomly selected. Additionally, the fact that women who did not return to immunization clinics could not be interviewed, was a possible source of selection bias. This study was not based on observational data, but on self-reports which is prone to courtesy bias. Use of the Bowser and Hill’s framework for D&A during childbirth did not permit exploitation of professional and structural/systemic definitions of mistreatment during childbirth as defined by Bohren et al. [7]

The survey was conducted in a hospital setting and by health workers, thus introducing courtesy bias due to women’s fear of aftermaths and non-confidentiality of their responses. Normalization of D&A during childbirth and fear of indicting health workers may have led to socially desirable responses. These could have resulted in underestimation of D&A during childbirth in both facilities. To mitigate this, different domains of D&A during childbirth were thoroughly explained and women were encouraged to give sincere responses. They were also assured of confidentiality and non-penalization for their responses.

Recall bias could have affected responses, given that the study was conducted from childbirth to 14 weeks thereafter. Conversely, a short recall period may lead to underreporting as women may be too fatigued to accurately relate their birth experiences. A longer recall period affords more time for women to recollect and correctly report their experiences. On the other hand, women may tend to forget these experiences as the newborn develops well [45]. Involvement of the researcher and other health providers in data collection may have introduced observer bias possibly underestimating the burden of D&A during childbirth. The researchers, however, were not directly involved in maternity care and were trained on data collection techniques. Finally, this study was conducted in only two facilities offering specialist care and given the age and educational profiles of the participants, it may not be generalizable to lower levels of care and the general population.

## Conclusion

A greater proportion of women who gave birth in the public hospital experienced at least one form of D&A during childbirth compared to those in the private one. Prevalence, however, was very high in both facilities. Abandonment/neglect and non-consented care were the two commonest forms of D&A in the private hospital, while in the public hospital abandonment and non-dignified care were the most frequent forms. Contextual barriers to respectful and non-abusive care of women during childbirth in both public and private health care settings need much more focus to be identified and addressed. We recommend adoption of a systems thinking approach in curbing various forms of D&A during childbirth highlighted in this study.

## Supplementary Information

**Additional file 1.**

**Additional file 2.**

**Additional file 3.**

## Data Availability

The datasets used and/or analyzed during the current study are available from the corresponding author on reasonable request.

## Abbreviations

AOR: Adjusted Odds ratio
CI: Confidence Interval
COR: Crude Odds ratio
D&A: Disrespect and Abuse
MNH: Maternal and New born Health
PCA: Principal Component Analysis
RMC: Respectful Maternity Care
SD: Standard Deviation
SBA: Skilled Birth Attendants
SPSS: Statistical Package for Social Sciences
WHO: World Health Organization
