# Supplementary material for: Does disrespect and abuse during childbirth differ between public and private hospitals in Southeast Nigeria
Source: BMC Pregnancy Childbirth. 2021 Dec 31;21:852. doi: 10.1186/s12884-021-04298-z (PMC8719415; doi:10.1186/s12884-021-04298-z)
Supplement: Supplementary file 3 — Additional file 3. [file 12884_2021_4298_MOESM3_ESM.docx]

Dear Editor,

Firstly, the authors wish to appreciate your patience and goodwill in repeatedly reviewing our manuscript. We sincerely apologise for the many flaws on the manuscript that you have painstakingly reviewed and corrected. We have carefully gone through and addressed the issues raised and we earnestly hope that the manuscript is now in an acceptable form. Once again, thank you so much for the rich learning process that this long peer review has afforded us as authors. .

RESPONSE TO COMMENTS FROM THE EDITOR

Results

The issue is that with a cOR 0.21; 95% CI 0.07-0.63, the interpretation should be that pin public hospital less women had their pubic hair shaved without consent than in the private hospital

Response: The authors appreciate this correction and have effected it (L230-31):

In the public hospital, less women 4 (1.3%) had their pubic hair shaved without consent than in the private hospital 18 (5.8%) [cOR 0.21; 95% CI. 0.07, 0.63]

1. Discussion

a. This is true, but at the same time these data were also rather high in the private hospital. That together points to the fact that the population in these two hospitals are very different from the distribution of education and wealth quartiles in the general population in Niigeria

Response: The authors have also highlighted this line of thought in the paper (L310-312)

……………..However, the population in these two hospitals may be different from the distribution of education and wealth quartiles in the general population in Nigeria.^9,33^

b. In fact receiving more women with complications should make staff even more friendly than in just uncomplicated women.

Response: The authors have included this in the discussion (L319-321)

c. I have no idea what this is?

Response: The authors have corrected this to read ‘…….the commonest type of D&A meted out to women during childbirth’

d. This isn’t discussion, this is just repetition of your results omit and start next sentence with In contrast with our findings

Response: This correction has been effected (L331)

e. This is unacceptable formulation, I hope you did not “use your participants”! If I understand this sentence you probably mean: An explanation could be that women in those studies were interviewed earlier after birth than in ours.””

Response: The authors apologize for this wrong choice of words and have promptly made the corrections as suggested by the Editor (L315).

f. Although lines 314-317 suggest to be findings from your study, you refer to another study and again beware of just repeating your findings

Response: The authors have revised and deleted repetitions (L342-44)

g. Unnecessary repetitions

Response: The authors apologise and have promptly deleted them (346-49)

h. Is fairness the opposite of discrimination?

Response: The authors have used non-discrimination in place of fairness (L349)

i. Here you can also argue for the opposite: women forget the nasty things the londer time has passed

Response: The authors had reflected this in the previous revision in the statement below:

‘Alternatively, a different school of thought opines that forgetfulness may be more likely if there is a longer duration between the experience and time of the survey’

However for ease of clarity, we have revised this to read:

Alternatively, a different school of thought opines that women may tend to forget the nasty birth experiences the longer the time passed between the experience and the survey

j. Is “another study (ref 22) in next sentence from the same countries as study ref by 44? Only then you could state “Another”, otherwise it would be “A study

Response: This correction has been effected (L358)

k. Did study with ref 22 also observe women-provider interaction. If yes “similar” is OK, if not it is not similar

Response: Response: The authors have corrected and reworded this statement (L360-61)

l. Here you ref to 26 and then “in this study” becomes unclear which study!

Response: The authors have corrected and reworded this statement (L360-61)

Observation of women-provider interactions by trained observers in an Ethiopian study found a prevalence of 74.8%, nonetheless, only 22% of the women reported D&A during childbirth.

m. Asking health care providers about this is a completely different issue and should not be in this discussion.

Response: The authors were only trying to critique the estimates from this published review however the part on health providers has been removed from the discussion

n. Older than 19, education > ninth gr and sec, probably indicate less D&A, longer labour, parity<4, self-reported depression, birth by CS, absence of support related with more or less D&A?p

Response: The authors have revised thus:

Response: In other studies, being older than 19 years, having no formal education, at least ninth grade and secondary education, self-reported depression, absence of support persons during childbirth, longer labour duration, and birth via Caesarean section were more likely to experience D&A during childbirth while parity (more than four births) reduced the likelihood

o. This sentence just repeats your findings and the next sentence how you can make a similar message in one sentence

Response: The sentence has been removed

p. What is childbirth without recognizing?

Response: The authors have now revised to read thus (L403):

Women who reside in rural areas tend to be poorer, less educated, less equipped with materials for childbirth, less aware of their rights and thus more prone to experience D&A during labour and childbirth without recognizing i**t**
